# Safety and efficacy of the keystone and rhomboid flaps for immediate reconstruction after wide local excision of non-head and neck melanomas

**DOI:** 10.1186/s12957-016-1019-x

**Published:** 2016-10-19

**Authors:** Mona Taleb, Lydia Choi, Steve Kim

**Affiliations:** 1 Department of Surgery at the Wayne State University School of Medicine, Detroit, MI USA; 2 Karmanos Cancer Center, 4100 John R, Mail Code HW04HO, Detroit, MI 48201 USA

**Keywords:** Melanoma, Margins, Reconstruction, Flap, Rhomboid, Keystone

## Abstract

**Background:**

After wide local excision of cutaneous melanoma, large defects not amenable to simple primary closure are often covered with skin grafts. We report our experience using the rhomboid and keystone flaps to immediately close large axial and extremity wounds after potentially curative surgery for non-head and neck melanomas.

**Methods:**

Between January 2011 and September 2016, demographic, operative, pathologic, and outcome data were prospectively collected on 60 patients who underwent wide local excision of melanoma followed by immediate flap reconstruction. Flaps were of either rhomboid or keystone type. Chi-square analysis was used to compare relationships between factors.

**Results:**

All procedures were done by the senior author and as outpatient surgery. No patient required a surgical drain unless they were undergoing concomitant radical regional node dissection. Flap separation (arbitrarily defined as a >5-mm dehiscence of the suture line) occurred in 16/61 patients (26 %). No patient had flap loss. The risk of flap morbidity was significantly higher if the primary tumor was on the distal extremity—10 of 24 patients (42 %), all with keystone flaps—than if it was on the trunk or the proximal extremity (6/37 patients, 16 %), *p* = 0.04. There were no margins positive for either invasive or in situ melanoma in the entire cohort.

**Conclusions:**

Simple transposition flaps can successfully cover large defects after melanoma excision without the need for skin grafting. Keystone flaps in the distal extremity are more prone to separation, but this is minor and does not result in flap loss. There is minimal risk of a positive margin requiring flap takedown and a second re-excision.

## Background

After wide local excision of cutaneous melanoma, large defects not amenable to simple primary closure are often covered with skin grafts. Although expedient and highly functional, grafting does result in two surgical sites (donor and recipient) and a potentially less satisfactory cosmetic outcome. An alternative is the use of transposition flaps to close large wounds. We report our experience using the rhomboid and keystone flaps to immediately close large axial and extremity wounds after potentially curative surgery for non-head and neck melanomas. We reviewed our experience for the rate of complications as well as the incidence of positive margins requiring flap takedown and re-excision.

## Methods

We examined a prospectively collected database of all patients undergoing cutaneous melanoma excision by the senior author between January 2011 and September 2016 at the Karmanos Cancer Center (Detroit, MI). During this time period, 61 patients underwent excision and immediate reconstruction with a skin flap. Reconstruction was performed with either keystone or rhomboid flaps using previously described techniques [1, 2]. All cases were performed as outpatient surgery. No special postoperative dressings, wound appliances, or extremity immobilization devices were utilized. Patients were told to limit weight-bearing only to necessary activities of daily living until the first postoperative visit. Demographics, operative, pathologic, and outcome data formed the database. The main outcome measures were flap separation (arbitrarily defined as a >5-mm dehiscence of the suture line), margin status, and local tumor recurrence. All chart reviews, data recordings, and analyses were carried out after IRB approval and abiding by federal and institutional HIPAA guidelines. Photographic consent was obtained as part of the consent for surgery. Statistical analysis of the data was performed using the SPSS software. Chi-square analysis of the data was used for comparison of the various factors.

## Results

**Fig. 1:**
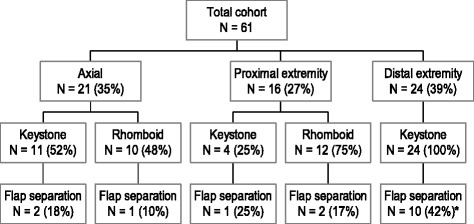
Primary tumor locations and type of flap reconstructions are charted. Total flap separation rate (as defined as a dehiscence >5 mm) was 16/60 (27 %). *Asterisk* denotes that the risk of flap separation was significantly higher for reconstructions on the distal extremity when compared to those on the proximal extremity or axial locations (*p* = 0.02)

There were 35 women and 26 men with median/mean age of 53/55 years (range 22–91 years). Median/mean Breslow depth was 2.0 mm/3.0 mm (range 0.27–22.0 mm), and 16 patients (26 %) had ulcerated lesions. All lesions ≥1 mm in depth were excised with 2-cm margins while those <1 mm were excised with 1-cm margins. Median/mean total excised area was 22.5/24.8 cm^2^ (range 5–70 cm^2^). Sentinel lymph node biopsy was performed in 53 patients (87 %) and was positive in 15 patients (28 %) (Table 1). One patient had clinically positive inguinal nodes at presentation and went directly to radical lymphadenectomy. There were 21 axial lesions, 16 proximal extremity lesions (defined as above the elbow or knee), and 24 distal extremity lesions (below the elbow or knee). Thirty-nine patients (64 %) had keystone flap reconstruction (11 axial, 4 proximal extremity, 24 distal extremity) and 22 patients (37 %) had rhomboid flap (10 axial and 12 proximal extremity). Flap separation occurred in 16 patients (27 %). No patient had flap loss. Flap type (rhomboid vs keystone) was not associated with a higher risk of flap separation; however, there was a greater risk of this event if the primary tumor was on the distal extremity—10 of 24 patients (42 %), all with keystone flaps—than if it was on the trunk or the proximal extremity (6/37 patients, 16 %), *p* = 0.04 (Fig. 1). There were no margins positive for either invasive or in situ melanoma in the entire cohort. One patient with dysplastic nevus syndrome had an incidental, discontinuous, dysplastic nevus at a margin, and this required re-excision. At a median/mean follow-up of 3.2 years, two locoregional recurrences were noted (3 %). A 76-year-old woman with a pT3aN0 calf melanoma developed an in-transit metastasis 3 cm above the keystone flap 16 months after excision. Another 46-year-old woman with a pT3aN1 calf melanoma had a local recurrence under the keystone flap suture line at 1 year and then subsequently was found to have multiple in-transit metastases throughout the leg 2 months later.

**Table 1:** Summary of patient, tumor, and operative data (see also Fig. 1)

Number of patients	61
Gender	
Male	26 (43 %)
Female	35 (57 %)
Age (years)	
Median	53
Mean	55
Range	22–91
Breslow depth (mm)	
Median	2.0
Mean	3.0
Range	0.27–22.0
Ulceration	
Present	16 (26 %)
Absent	45 (74 %)
Total excised area (cm^2^)	
Median	22.5
Mean	24.8
Range	5–70
Sentinel lymph node biopsy	
Total number of SLNB	53
Positive SLNB	15 (28 %)

## Discussion

**Fig. 2:**
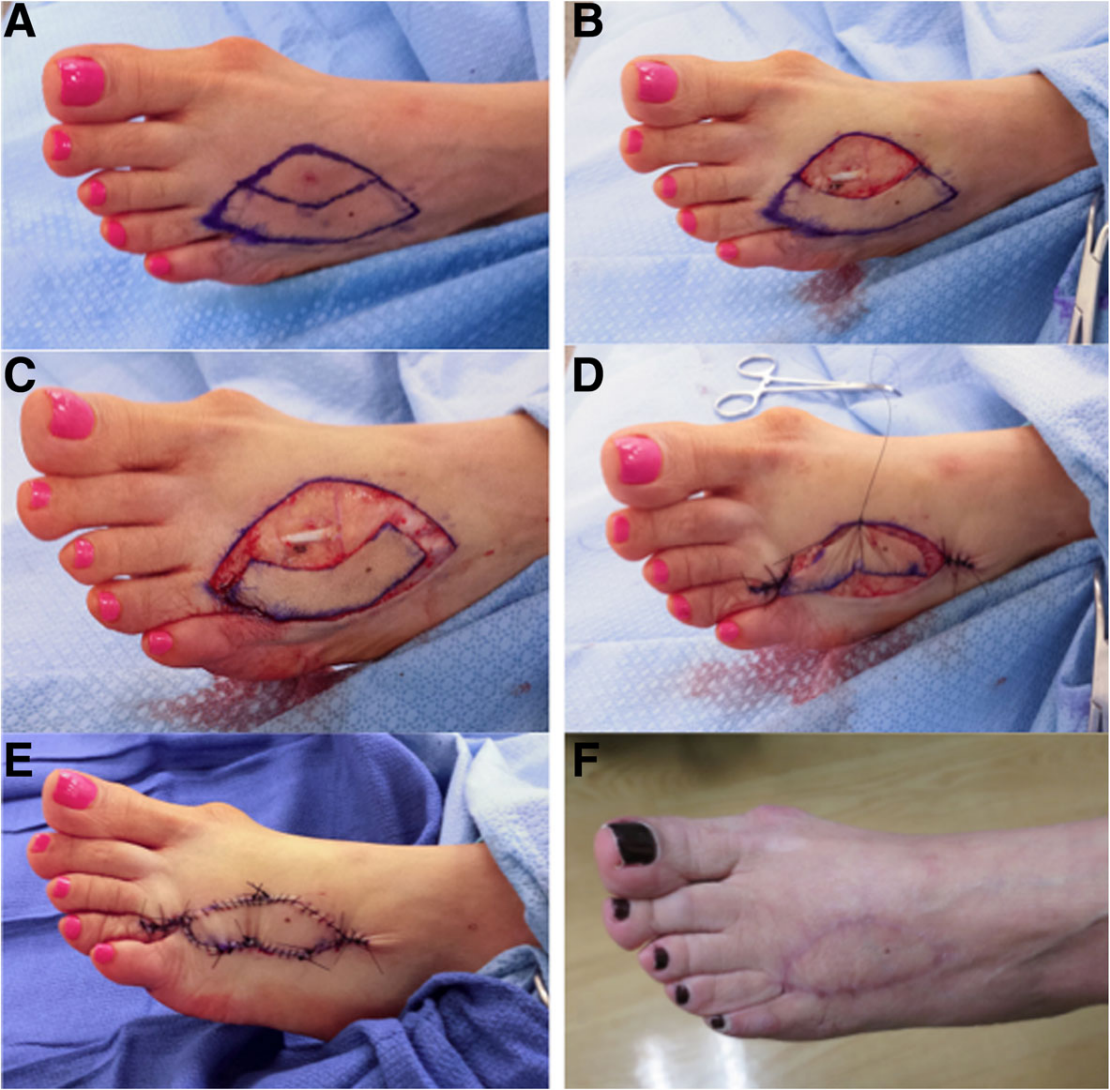
Keystone flap closure of a melanoma excision defect on the left foot. The patient presented with a T1a lesion on the dorsum of her left foot (**a**). Appropriate wide excision has been performed (**b**). A keystone flap has been created at the lateral aspect of the original excision bed (**c**) and then transposed medially (**d**) to close the defect (**e**). Postoperative appearance at 8 months is shown (**f**)

**Fig. 3:**
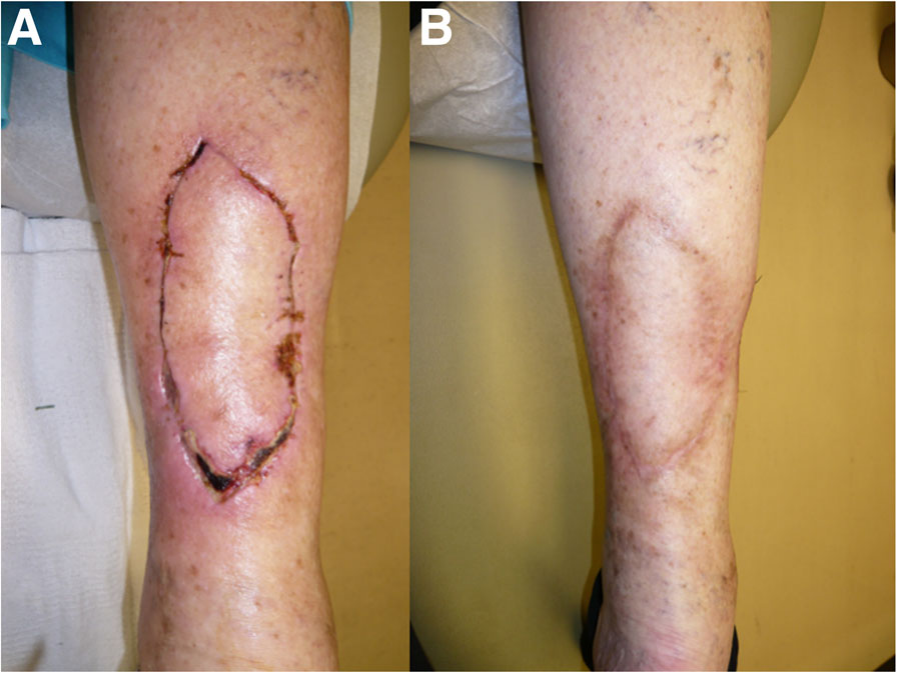
Partial dehiscence of a melanoma excision defect on the posterior calf after keystone flap reconstruction (**a**). Postoperative appearance at 1 year (**b**)

In our cohort of 61 patients who underwent immediate transposition flap reconstruction of large defects resulting from curative excision of cutaneous melanoma, the rate of flap separation was highest in the distal extremity (42 %). This might be expected since skin and subcutaneous tissue laxity is frequently the least generous below the elbow and knee. In our study, all reconstructions in the distal extremity were performed with keystone flaps. The keystone flap and its modifications are techniques for closing elliptical defects previously thought to require skin grafting [2, 3]. The method relies on creating a “keystone”-shaped flap adjacent to the elliptical excision bed, thus creating an even larger ellipse. Two adjacent V-Y advancement closures are then performed at the ends of the larger ellipse, theoretically resulting in less tension when the middle part of the keystone is advanced to close the initial excision bed (Fig. 2). There has been some recent controversy over the scientific basis and mechanism behind the concept of this technique [4, 5]. In our experience, however, we feel that this is an excellent alternative to skin grafting for closure of large defects after melanoma excision. We attribute our relatively high rate of morbidity with this technique mostly to our low threshold for defining flap separation (any degree of dehiscence >5 mm was counted as an event). However, none of the dehiscences required re-intervention or resulted in flap loss, and closure by secondary intention usually resulted in a good cosmetic outcome (Fig. 3).

As a final point, some authors have advocated that reconstruction of skin defects after cancer excision should be done only after final pathologic diagnosis has been rendered, especially in esthetically sensitive areas and/or with complex reconstructions since this minimizes the amount of tissue initially excised [6]. This obviates the need for flap takedown should further excision be required due to a positive margin. Unfortunately, routine hematoxylin and eosin frozen section assessment of margins for melanocytic lesions has been found to be notoriously inaccurate [7, 8]. In our cohort, however, no margins were positive for invasive melanoma or melanoma in situ on permanent section. Only one re-excision was necessary due to an incidental, discontinuous, high-grade dysplastic nevus in a patient with dysplastic nevus syndrome. Suture line recurrence occurred in only one patient, and she had initial advanced disease (T3N1) and subsequent rapid and widespread in-transit and skin metastases. Hence, we conclude that it is safe and justified to perform immediate reconstruction of defects after wide local excision, since there is a very low risk of a positive margin requiring re-excision and flap takedown. This is concordant with other reports that have demonstrated an infrequent need (2 %) for melanoma re-excision due to positive margins and/or early local tumor recurrence [9].

Our study has certain limitations. It is of relatively small size (*N* = 61) and involved the experience of only a single surgeon (although alternatively, there might also be an advantage to this since surgical technique was consistent in all cases). Despite these, it appears to validate the results of other reports that show good long-term cosmetic outcomes and low margin positivity rate when immediate flap reconstruction is performed after wide local excision of melanoma [2, 3, 9].

## Conclusions

The keystone and rhomboid flaps are useful techniques to close wounds that are not amenable to primary repair. The former is associated with increased but mild morbidity when used in the distal extremity. Positive margins after wide local excision of non-head and neck melanomas are a rare event, justifying immediate flap reconstruction of the skin defect when necessary.
